# SART-Type Image Reconstruction from Overlapped Projections

**DOI:** 10.1155/2011/549537

**Published:** 2010-09-05

**Authors:** Hengyong Yu, Changguo Ji, Ge Wang

**Affiliations:** ^1^Division of Radiologic Sciences, Department of Radiology, Wake Forest University Health Sciences, Winston-Salem, NC 27157, USA; ^2^Biomedical Imaging Division, VT-WFU School of Biomedical Engineering and Sciences, Wake Forest University Health Sciences, Winston-Salem, NC 27157, USA; ^3^Biomedical Imaging Division, VT-WFU School of Biomedical Engineering and Sciences, Virginia Tech, Blacksburg, VA 24061, USA

## Abstract

To maximize the time-integrated X-ray flux from multiple X-ray sources and shorten the data acquisition process, a promising way is to allow overlapped projections from multiple sources being simultaneously on without involving the source multiplexing technology. The most challenging task in this configuration is to perform image reconstruction effectively and efficiently from overlapped projections. Inspired by the single-source simultaneous algebraic reconstruction technique (SART), we hereby develop a multisource SART-type reconstruction algorithm regularized by a sparsity-oriented constraint in the soft-threshold filtering framework to reconstruct images from overlapped projections. Our numerical simulation results verify the correctness of the proposed algorithm and demonstrate the advantage of image reconstruction from overlapped projections.

## 1. Introduction

Since the first computed tomography (CT) scanner was made [1], all the commercial scanners have been employing the X-ray source with a single small focal spot, which can be modeled as a point source. In micro-CT and even nano-CT applications, the reduced X-ray focal spot size becomes increasingly a limiting factor to achieve contrast and temporal resolution targets. To address this issue, our group recently proposed to use a line-shaped X-ray source so that more photons can be generated in a given data acquisition interval [2]. In this context, the X-ray source can be modeled as a line-segment, which can be further discretely modeled as an array of points [3]. In single source CT scanners, contrast resolution is limited by the finite focal-spot size necessary to generate a sufficient number of X-ray photons, and temporal resolution is limited by the time taken to acquire sufficiently many projections over a full-scan or half-scan angular range. Since a line source covers a wide angular range per view, the number of photons is increased to radiate an object to be reconstructed. Therefore, use of a line-shaped X-ray source or a multiple source array is a candidate scheme to balance spatial, contrast and temporal resolution.

 Interestingly, the technology of field-emission X-ray sources based on carbon nanotubes (CNT) is a recent invention with several intrinsic advantages over conventional X-ray tubes. To maximize the time-integrated X-ray flux from multiple sources and improve the signal-to-noise ratio (SNR), multiplexing was used, where multi-source are excited with different temporal modulations [4]. If two or more sources are simultaneously fired, X-ray photons reach the same detector together for any single measurement, and one would not be able to identify which photon comes from which source. To unmix the signals from various sources, several projections can be collected at different time instants for the same view [5]. Due to the limited readout speed and mixed signals, there seems little advantage to use multiplexing configuration in terms of contrast and temporal resolution. In collaboration with Dr. Otto Zhou's group at University of North Carolina, we are developing a novel multi-source micro-CT system. In our system, we plan to fire a multi-source system simultaneously and acquire multiple projections at any viewing angle on nonoverlapped segments of a shared detector array [6]. With recent developed compressive sampling (CS) techniques [7, 8], we are also working to improve temporal resolution and reduce radiation dose from a limited number of nonoverlapped projections [9]. 

 Here, we consider how to reconstruct an image from overlapped projections. Previously, our group developed a generalized simultaneous algebraic reconstruction technique (SART) algorithm to reconstruct an image from data collected with an X-ray line source [2]. Assuming that the differences between measured and predicted projections from various source points have equal weights, these differences are then backprojected to different X-ray source points in the SART framework. However, this algorithm converges to the least square solution that is not necessarily the true image. In our simulation, the reconstructed images sometimes suffered from blurring [2]. Then, our group developed a CS-based algorithm to solve this problem [3]. The algorithm is implemented in a projection onto convex sets (POCS) framework and employed a steepest gradient searching strategy. Although this algorithm often converges to the true image, its convergence speed is rather slow. Because the SART framework has an excellent convergence behavior especially when the ordered subset (OS) format is applied, in this paper we will develop a SART-type algorithm for image reconstruction from overlapped projections.

 The paper is organized as follows. In the next section, we will formulate the overlapped projection model, which is not a sum of line integrals and quite different from that in the single-source case. In the third section, we will design a SART-type reconstruction algorithm for image reconstruction from overlapped projections. In the fourth section, we will report numerical results. In the last section, we will discuss related issues and conclude the paper. 

## 2. Imaging Model

### 2.1. Nonoverlapped Projection Model

**Figure 1:**
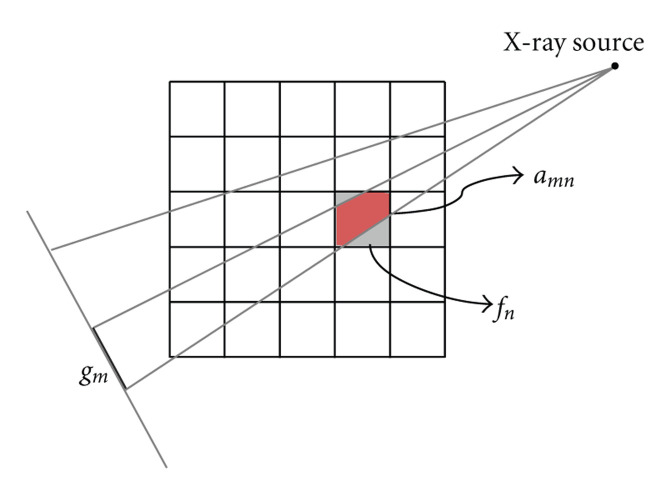
Projection model of a discrete image in fan-beam geometry.

For CT reconstruction, a two-dimensional digital image can be expressed as **f** = (*f*_*i*,*j*_) ∈ ℝ^*I*^ × ℝ^*J*^, where the index 1 ≤ *i* ≤ *I* and 1 ≤ *j* ≤ *J* are integers. Define

(1) $$f_{n}=f_{i,j},\;n=\left(i-1\right)\times J+j,$$

with 1 ≤ *n* ≤ *N* and *N* = *I* × *J*, we have the image in a vector representation **f** = [*f*_1_,*f*_2_,...,*f*_*N*_]^*T*^ ∈ ℝ^*N*^. In this paper, we will use both the signs *f*_*i*,*j*_ and *f*_*n*_ for convenience. Let **g** = [*g*_1_,*g*_2_,...,*g*_*M*_]^*T*^ ∈ ℝ^*M*^ be a measured vector with *M* being the product of the number of projections and the number of detector elements. They are linked by the following linear system:

(2) g=Af,

where **A** = (*a*_*m*,*n*_) ∈ ℝ^*M*^ × ℝ^*N*^ is a measurement matrix. In a typical fan-beam geometry, the *n*th pixel can be viewed as a rectangular region with a constant value *f*_*n*_, the *m*th measured datum *g*_*m*_ as an integral of partially covered pixel areas by a narrow beam from an X-ray source to a detector element which are weighted by the corresponding X-ray linear attenuation coefficients. Thus, the component *a*_*m*,*n*_ in (2) denotes the intersection area between the *n*th pixel and the *m*th fan-beam ray (Figure 1). While the whole matrix **A** represents the forward projection, **A**^*T*^ implements the backprojection.

### 2.2. Overlapped Projection Model

While the imaging model (2) is valid for a single-source system, it cannot be used for multi-source-generated projections. The reason is that the measured data in (2) has been postprocessed by a logarithmic operation. In other words, we must model the raw data directly. For a multi-source system with *Q* sources, assuming that the *q*th source emits *I*_*q*_ photons towards each detector element. According to Beer's law, we have the following imaging model:

(3) $$p=\overset{Q}{\underset{q=1}{\sum }}I_{q}e^{-sA^{q}f},$$

where **A**^*q*^ is the system matrix defined in Section 2.1 for the *q*th source, *s* a constant to normalize the area model (Figure 1) for the measurement matrix to the line integral model, and *e*^−*s ***A**^*q*^**f**^ is a vector whose element is the exponential function of the corresponding element of −*s ***A**^*q*^**f**. In practice, we can approximate *s* as the reciprocal of the average width of an X-ray path in an object to be reconstructed. If we assume that all the X-ray sources have the same intensities, *I*_1_ = *I*_2_ = ⋯ = *I*_*q*_ = *I*_0_, we have

(4) $$\tilde{p}=\frac{p}{I_{0}}=\overset{Q}{\underset{q=1}{\sum }}e^{-A^{q}\tilde{f}},$$

where the constant *s* is absorbed by $\tilde{f}=sf$, and 1/*I*_0_ absorbed by $\tilde{p}=\tilde{p}/I_{0}$. The key task in this paper is to reconstruct $\tilde{f}$ from $\tilde{p}$ and known measurement matrices **A**^*q*^.

## 3. Reconstruction Algorithm

### 3.1. Single-Source Algorithm

In the past decade, our group studied a block-iterative (BI) or ordered-subset (OS) version of a general Landweber scheme [10], of which the SART and OS-SART [11] are special cases, for minimization of a weighted least square functional in the real/complex space, and proved its convergence under quite general conditions [12, 13]. The SART or OS-SART technology has been widely used in the CT field. Particularly, for the system (2) the SART-type solution is given by [11]

(5) $$f_{n}^{k}=f_{n}^{k-1}+\lambda^{k}\frac{1}{a_{+n}}\overset{M}{\underset{m=1}{\sum }}\frac{a_{m,n}}{a_{m+}}\left(g_{m}-A_{m}f^{k-1}\right),$$

where $a_{+n}=\sum_{m=1}^{M}a_{m,n}>0$, $a_{m+}=\sum_{n=1}^{N}a_{m,n}>0$, **A**_*m*_ is the *m*th row of **A**, *k* the iteration index, and 0 < *λ*^*k*^ < 2 a free relaxation parameter. Let Λ^+*N*^ ∈ ℝ^*N*^ × ℝ^*N*^ be a diagonal matrix with Λ_*n*,*n*_^+*N*^ = 1/*a*_+*n*_ and Λ^*M*+^ ∈ ℝ^*M*^ × ℝ^*M*^ be a diagonal matrix with Λ_*m*,*m*_^*M*+^ = 1/*a*_*m*+_, (5) can be rewritten as

(6) $$f^{k}=f^{k-1}+\lambda^{k}\left(\Lambda^{+N}A^{T}\Lambda^{M+}\left(g-Af^{k-1}\right)\right).$$

Note that for all the iteration index *k*, Λ^*M*+^, Λ^+*N*^ and **A** remain unchanged.

### 3.2. Multisource Algorithm

Since (4) is a non-linear equation, there is no analytic solution to this problem. Here, we will find a solution in the SART framework summarized in the proceeding subsection. Our strategy is to linearize the equation and approximate the solution successively. Denote the approximate solution for (4) as ${\tilde{f}}^{k}$ after *k* iterations, we have

(7) $$\tilde{p}=\overset{Q}{\underset{q=1}{\sum }}e^{-A^{q}({\tilde{f}}^{k}+\tilde{f}-{\tilde{f}}^{k})}=\overset{Q}{\underset{q=1}{\sum }}e^{-A^{q}({\tilde{f}}^{k}+\Delta {\tilde{f}}^{k})}=\overset{Q}{\underset{q=1}{\sum }}\left(e^{-A^{q}{\tilde{f}}^{k}}⊙e^{-A^{q}\Delta {\tilde{f}}^{k}}\right),$$

where $\Delta {\tilde{f}}^{k}$ is the error image, and “⊙” represents the component-wise multiplication of vectors of the same size. Then, $e^{-A^{q}\Delta {\tilde{f}}^{k}}$in (7) can be expanded in a Taylor series

(8) $$e^{-A^{q}\Delta {\tilde{f}}^{k}}=\overset{\infty }{\underset{t=0}{\sum }}\frac{{\left(-1\right)}^{t}{\left(A^{q}\Delta {\tilde{f}}^{k}\right)}^{t}}{t!},$$

where ${\left(A^{q}\Delta {\tilde{f}}^{k}\right)}^{t}=\left(A^{q}\Delta {\tilde{f}}^{k}\right)⊙\left(A^{q}\Delta {\tilde{f}}^{k}\right)\cdots .$ Substituting the 1st order approximation of $e^{-A^{q}\Delta {\tilde{f}}^{k}}$ into (7), we have

(9) $$\begin{array}{cc} \tilde{p} & \approx \overset{Q}{\underset{q=1}{\sum }}\left(e^{-A^{q}{\tilde{f}}^{k}}⊙\left(1-A^{q}\Delta {\tilde{f}}^{k}\right)\right)\\ & =\overset{Q}{\underset{q=1}{\sum }}e^{-A^{q}{\tilde{f}}^{k}}-\overset{Q}{\underset{q=1}{\sum }}\left(e^{-A^{q}{\tilde{f}}^{k}}⊙\left(A^{q}\Delta {\tilde{f}}^{k}\right)\right)\\ & =\overset{Q}{\underset{q=1}{\sum }}e^{-A^{q}{\tilde{f}}^{k}}-\left(\overset{Q}{\underset{q=1}{\sum }}\left(E^{q,k}A^{q}\right)\right)\left(\Delta {\tilde{f}}^{k}\right), \end{array}$$

where **E**^*q*,*k*^ ∈ ℝ^*M*^ × ℝ^*M*^ be a diagonal matrix with $E_{m,m}^{q,k}=e^{-A_{m}^{q}{\tilde{f}}^{k}}$. Equation (9) can be rewritten as

(10) $${\tilde{g}}^{k}=B^{k}\Delta {\tilde{f}}^{k},$$

where

(11) $$\begin{array}{c} {\tilde{g}}^{k}=\overset{Q}{\underset{q=1}{\sum }}e^{-A^{q}{\tilde{f}}^{k}}-\tilde{p},\\ B^{k}=\overset{Q}{\underset{q=1}{\sum }}\left(E^{q,k}A^{q}\right). \end{array}$$

Since the projection errors ${\tilde{g}}^{k}$ from the involved sources are known, (10) can be understood as a linear system of $\Delta {\tilde{f}}^{k}$ with a measurement matrix **B**^*k*^. 

 Because (10) has the same structure as that of (2), we can use the SART-type formula (6) to solve for $\Delta {\tilde{f}}^{k}$. Let Λ^+*N*,*k*^ ∈ ℝ^*N*^ × ℝ^*N*^ be a diagonal matrix with

(12) $$\Lambda_{n,n}^{+N,k}=\frac{1}{\sum_{m=1}^{M}b_{m,n}^{k}},$$

and Λ^*M*+,*k*^ ∈ ℝ^*M*^ × ℝ^*M*^be a diagonal matrix with

(13) $$\Lambda_{m,m}^{M+,k}=\frac{1}{\sum_{n=1}^{N}b_{m,n}^{k}},$$

we have the SART-type solution for $\Delta {\tilde{f}}^{k}$

(14) $$\Delta {\tilde{f}}^{k,l}=\Delta {\tilde{f}}^{k,l-1}+\lambda^{k,l}\left(\Lambda^{+N,k}{\left(B^{k}\right)}^{T}\Lambda^{M+,k}\left({\tilde{g}}^{k}-B^{k}\Delta {\tilde{f}}^{k,l-1}\right)\right),$$

where *l* is the iteration index and *λ*^*k*,*l*^ the relax parameter. Since

(15) $$\begin{array}{cc} {\left(B^{k}\right)}^{T} & ={\left(\overset{Q}{\underset{q=1}{\sum }}\left(E^{q,k}A^{q}\right)\right)}^{T}=\overset{Q}{\underset{q=1}{\sum }}{\left(E^{q,k}A^{q}\right)}^{T}\\ & =\overset{Q}{\underset{q=1}{\sum }}\left({\left(A^{q}\right)}^{T}{\left(E^{q,k}\right)}^{T}\right)=\overset{Q}{\underset{q=1}{\sum }}\left({\left(A^{q}\right)}^{T}E^{q,k}\right), \end{array}$$

Equation (14) can be rewritten as

(16) $$\begin{array}{cc} \Delta {\tilde{f}}^{k,l} & =\Delta {\tilde{f}}^{k,l-1}+\lambda^{k,l}(\Lambda^{+N,k}\left(\overset{Q}{\underset{q=1}{\sum }}\left({\left(A^{q}\right)}^{T}E^{q,k}\right)\right)\\ & \;\;\;\;\;\;\;\times \Lambda^{M+,k}\left({\tilde{g}}^{k}-B^{k}\Delta {\tilde{f}}^{k,l-1}\right))\\ & =\Delta {\tilde{f}}^{k,l-1}+\lambda^{k,l}(\overset{Q}{\underset{q=1}{\sum }}((\Lambda^{+N,k}{\left(A^{q}\right)}^{T}E^{q,k}\\ & \;\;\;\;\;\;\;\times \Lambda^{M+,k}\left({\tilde{g}}^{k}-\;B^{k}\Delta {\tilde{f}}^{k,l-1}\right)))\\ & =\Delta {\tilde{f}}^{k,l-1}+\lambda^{k,l}(\overset{Q}{\underset{q=1}{\sum }}(\Lambda^{+N,k}{\left(A^{q}\right)}^{T}\Lambda^{M+,k}\\ & \;\;\;\;\;\;\;\times E^{q,k}\left({\tilde{g}}^{k}\;-\;B^{k}\Delta {\tilde{f}}^{k,l-1}\right)))\\ & =\Delta {\tilde{f}}^{k,l-1}+\lambda^{k,l}(\overset{Q}{\underset{q=1}{\sum }}(\Lambda^{+N,k}{\left(A^{q}\right)}^{T}\Lambda^{M+,k}\\ & \;\;\;\;\;\;\;\times \left(e^{-A^{q}{\tilde{f}}^{k}}⊙\left({\tilde{g}}^{k}\;-\;B^{k}\Delta {\tilde{f}}^{k,l-1}\right)\right))) \end{array}$$

Once we have an approximation solution $\Delta {\tilde{f}}^{k,L}$ for $\Delta {\tilde{f}}^{k}$ after *L* iterations,we can update the reconstructed image by

(17) $${\tilde{f}}^{k+1}={\tilde{f}}^{k}+\Delta {\tilde{f}}^{k,L}.$$

For example, we can choose $\Delta {\tilde{f}}^{k,0}=0$ as the initial image and set *L* = 1 for one step iteration to approximate $\Delta {\tilde{f}}^{k}$, which results in a simplified algorithm

(18) $${\tilde{f}}^{k+1}={\tilde{f}}^{k}+\lambda^{k}\left(\overset{Q}{\underset{q=1}{\sum }}\left(\Lambda^{+N,k}{\left(A^{q}\right)}^{T}\Lambda^{M+,k}\left(e^{-A^{q}{\tilde{f}}^{k}}⊙{\tilde{g}}^{k}\right)\right)\right).$$

For numerical implementation, our SART-type reconstruction algorithm for image reconstruction from overlapped projections can be summarized in the following pseudocode:

(S.1.) initialize ${\tilde{f}}^{k}$ and *λ*^*k*^ for *k* : = 0;
(S.2.) compute ${\tilde{g}}^{k}$, Λ^+*N*,*k*^ and Λ^*M*+,*k*^;
(S.3.) initialize the error image $\Delta {\tilde{f}}^{k,1}:=0$;
(S.4.) for *q* = 1 to *Q* backproject the projection error;
  (S.4.1.) weight the projection error ${\tilde{g}}^{k}$ by $e^{-A^{q}{\tilde{f}}^{k}}$;
  (S.4.2.) weight the projection error ${\tilde{g}}^{k}$ by Λ^*M*+,*k*^;
  (S.4.3.) backproject the weighted projection error towards the *q*th X-ray source;
  (S.4.4.) weight the backprojected error image by Λ^+*N*,*k*^;
  (S.4.5.) add the backprojected image to $\Delta {\tilde{f}}^{k,1}$ by
    (19) $$\Delta {\tilde{f}}^{k,1}\;\text{:=}\;\Delta {\tilde{f}}^{k,1}+\Lambda^{+N,k}{\left(A^{q}\right)}^{T}\Lambda^{M+,k}\left(e^{-A^{q}{\tilde{f}}^{k}}⊙{\tilde{g}}^{k}\right);$$
(S.5.) update the current estimated image by

(20) $${\tilde{f}}^{k+1}\;\text{:=}\;{\tilde{f}}^{k}+\lambda^{k}\Delta {\tilde{f}}^{k,1};$$

(S.6.) set *k* : = *k* + 1;
(S.7.) go to (S.2) until the convergence criteria are satisfied;
(S.8.) scale the reconstructed image ${\tilde{f}}^{*}$ to obtain the final result $f^{*}={\tilde{f}}^{*}/s$.

In the above pseudo-code, (S.1.) initializes the iteration index *k*, the relax parameter *λ*, and the initial image ${\tilde{f}}^{0}$. In our numerical simulation, we always set *λ*^*k*^ = 1 and ${\tilde{f}}^{0}=0$. The outer loop (S.2)–(S.7) solves for $\tilde{f}$ successively. (S.2) precomputes several important intermediate variables to update an reconstructed image in the iteration step *k*. (S.3)-(S.4) computes the current error image for one step iteration according to (16). Because the backprojection operation for different X-ray sources in the inner loop (S.4) has the same structure, it can be implemented by calling a common procedure. (S.5) updates an reconstructed image. (S.6) updates the iteration index. In (S.7), the convergence criteria are checked. The stopping criteria for (S.7) can be the maximum iteration number is reached and/or the relative reconstruction error comes under a predefined threshold [14]. Finally, the reconstructed image f* is scaled by dividing with the constant *s*.

### 3.3. Sparsity Regularization

The conventional data acquisition is based on the Nyquist sampling theory, which states that for accurate reconstruction of a band-limited signal or image the sampling rate must be at least twice the highest frequency of the signal or image. However, the recently developed CS theory shows that a high-quality signal or image can be reconstructed from far fewer measurements than what is usually required by the Nyquist sampling theorem [7, 8]. In light of the work on solving the linear inverse problems with sparsity constraints by Daubechies et al. [14, 15], we recently adapted the single source SART to reconstruct an image from a limited number of projections subject to a sparsity constraint [16], and demonstrated that the sparsity constraint helped improve the quality of reconstructed images effectively and reduce the number of projections significantly. Using the same strategy described in our previous papers [16, 17], here we use the sparisty constraint to regularize the proposed multi-source SART algorithm. This can be done by adding a soft-threshold filtering step between (S.5) and (S.6) in the pseudo-code given in Section 3.2. Particularly, we have the following pseudo-code segment:

(SS.5.) perform a soft-threshold filtering operation for ${\tilde{f}}^{k+1}$;
  (SS.5.1.) compute the sparse transform;
  (SS.5.2.) estimate the optimal threshold;
  (SS.5.3.) perform the soft-threshold filtering;
  (SS.5.4.) perform the inverse sparse transform.

In the above pseudo-code, the sparse transform in (SS.5.1) can be either any invertible lossless compressible transform such as wavelet transform [16] and Fourier transform or uninvertible transforms such as discrete gradient transform (DGT) and discrete difference transform (DDT) [17]. For an uninvertible transform, the inverse sparse transform in (SS.5.4.) is in terms of pseudoinversion as we performed for DGT and DDT [17]. (SS.5.2.) determines an optimal threshold automatically using the projected gradient method for fast convergence [14]. In fact, we can omit (SS.5.2.) and specify any fixed filtering threshold. However, both the convergence speed and final result depend on the choice of the threshold. 

## 4. Numerical Simulation

**Figure 2:**
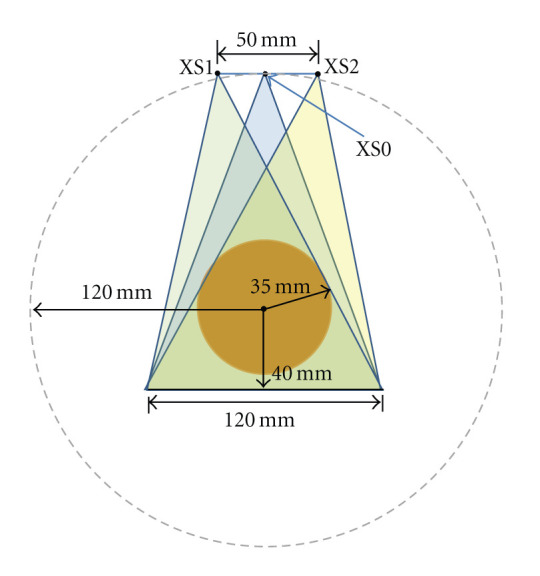
Geometrical configuration of a triple-source micro-CT system.

To verify the proposed SART-type algorithm for image reconstruction from overlapped projections, we implemented it in Matlab on a PC, with the computationally intensive segments coded in C and linked via the MEX mechanism. As illustrated in Figure 2, we simulated a triple-source fan-beam micro-CT system. In the system, the source XS0 is rotated on a circular scanning locus of radius 120 mm. The object was a modified Shepp-Logan phantom in a compact support with a radius of 35 mm. We used an equi-distance detector array of length 120 mm. The detector was perpendicular to the direction from the origin to the X-ray source XS0 that is 40 mm from the system origin. The detector array consisted of 500 elements. On the line through the X-ray source XS0 and parallel to the detector, we put two sources XS1 and XS2 being 25 mm apart from XS0 on its right and left sides, respectively. With this triple-source configuration, we simulated single-source (only XS0 was fired), dual-source (XS1 and XS2 fired simultaneously), and triple-source cases (XS0, XS1 and XS2 fired simultaneously). 

**Figure 3:**
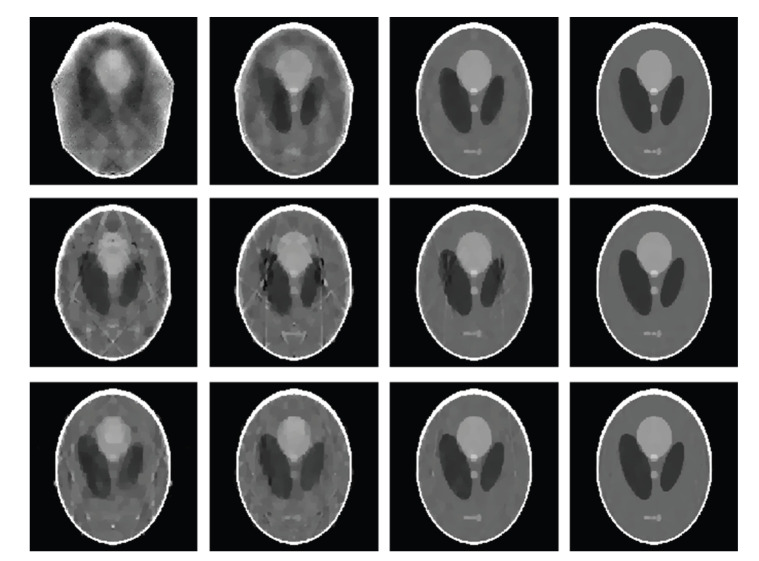
Reconstructed images from noise-free projections after 5000 iterations. Form the left to right columns, the projection numbers are 9, 11, 13 and 15, respectively. From the top to bottom rows, the source numbers are 1, 2, and 3, respectively. The display window is [0,0.5].

**Figure 4:**
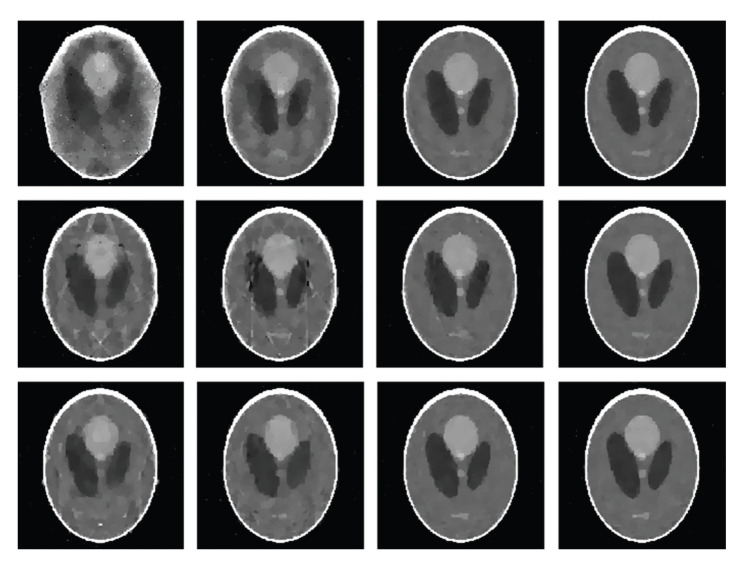
The counterparts of Figure 3 from datasets corrupted by Poisson noise.

 For each of our selected numbers of projections over a full-scan range, we first equiangularly acquired the corresponding projection dataset based on the aforementioned projection model in the single, dual, and triple source cases, respectively. Then, we reconstructed the images using our algorithm described in Section 3.2. In our simulation, the parameter *λ*^*k*^ in the SART formula (18) was set to 1.0, and the stopping criterion was defined as reaching the maximum iteration number 5000. Because the Shepp-Logan phantom is a piecewise constant function, its DGT and DDT are sparse. Hence, we also employed the sparsity regularization in terms of total difference minimization [17] and the threshold for filtering was automatically computed using the projected gradient method [14]. Figure 3 shows the reconstructed 256 × 256 images from 9, 11, 13, and 15 projections, respectively. For real-world applications, measurement noise is unavoidable. To test the stability of the proposed algorithm against data noise, we repeated the above reconstructions from projections corrupted by Poisson noise, assuming *I*_0_ = 5 × 10^4^ photons per detector element [18]. The results are in Figure 4, which indicate the stability of the proposed algorithm. 

## 5. Discussions and Conclusion

 In the CT field, the line integral model along an X-ray path has been widely used in consistency with Beer's law. However, it does not reflect the divergence due to the combination of the finite detector size and the source focal spot. As shown in Figure 1, we have assumed an area model for the X-ray path and normalized it for the multi-source imaging model. Our area model treats the X-ray path as a narrow fan-beam from the X-ray point source to the detector element, and we believe that the area model works better than the line model. Note that the proposed algorithm views the projection procedure as a matrix transform, both the area and line models can be handled by our algorithm. In other words, the proposed algorithm is independent of the imaging model as long as it is linear or can be transformed into a linear one. Additionally, to simplify the derivation and demonstrate our idea, we have assumed that the photon numbers emitting from all the sources to each detector are the same. In fact, these numbers may be different, and can be easily incorporated into our algorithm.

 As far as the convergence of the proposed algorithm is concerned, it should converge to the least square solution in the cases of either noise-free and noisy projections. The reason is that the proposed method is in the framework of the general Landweber scheme, whose convergence has been well studied under quite general conditions [12, 13]. When only a small number of projections are available, we can use some sparse constraints to steer the solution to the truth. However, the convergence speed of the current soft-threshold filtering technology is still slow although it has been accelerated using the projected gradient method [14]. In the future, we will employ more advanced techniques for a faster speed, which may include but not limited to optimizing the code, employing parallel computation, and developing new algorithms.

 In conclusion, we have developed a SART-type algorithm for image reconstruction from overlapped projections. The algorithm has been verified and demonstrated in the numerical simulation. Our methodology has a potential to support more flexible designs of multi-source CT/micro-CT systems for better contrast and temporal resolution.
